# Prevalence of malaria and associated factors among febrile children under 15 years at Bududa General Hospital, Eastern Uganda

**DOI:** 10.1186/s12936-024-05218-0

**Published:** 2025-02-07

**Authors:** Benson Okongo, Daisy Asiimwe, Clinton Olong, Enoch Muwanguzi, Robert Wagubi

**Affiliations:** 1 Department of Medical Laboratory Science, Mbarara University of Science and Technology, Mbarara, Uganda; 2 Department of Clinical Laboratories, Mbarara Regional Referral Hospital, Mbarara, Uganda; 3 Department of Pathology and Clinical Laboratories, Uganda Cancer Institute – Regional Cancer Centre, Gulu, Uganda

**Keywords:** Prevalence, Malaria, Factors, Febrile, Children, Uganda

## Abstract

**Background:**

A significant portion of malaria-related deaths occur in Africa, and Uganda is an endemic region where malaria remains a public health concern. This study aimed to determine the prevalence of malaria and its associated factors among febrile children under 15 years of age at Bududa General Hospital, Eastern Uganda.

**Methods:**

This cross-sectional study was conducted between April and June 2023. Informed consent was obtained from parents/guardians before 250 febrile children below 15 years were enrolled in this study. A structured questionnaire was administered to parents/guardians to collect sociodemographic characteristics and identify factors associated with malaria. Venous blood samples were collected from the children and screened for the presence of malaria parasitaemia using blood smear microscopy. The data collected were entered into an Excel spreadsheet and analysed using STATA version 14. Logistic regression models were used to determine the factors associated with malaria, and we considered ≤ 0.05 as the level of significance.

**Results:**

Out of the 250 study participants, the overall prevalence of malaria was 111(44.4%). Among the children who tested positive for malaria, 98 (88.3%) had *Plasmodium falciparum*, 11 (9.9%) had *Plasmodium malariae,* and (1.8%) had *Plasmodium ovale* infection. The mean parasite count was 21,951 parasites/µL of blood. The highest parasite count was 154,387 parasites/µL of blood, and the lowest count was 146 parasites/µL of blood. The prevalence rates of low, moderate, and high malaria parasitaemia were 46.8%, 28.0%, and 25.2%, respectively. In the multivariate analysis, the factors associated with malaria infections were older age; 1 to 5 years (p = 0.013), 6 to 10 years (p = 0.000), 11 to 15 years (p = 0.000), secondary education (p = 0.050), and no use of insecticide-treated bed nets (p = 0.002).

**Conclusion:**

The prevalence of malaria among febrile children in this study was high, with nearly half of the participants showing severe infections. Health education on the correct use of insecticide-treated mosquito nets should be prioritized to help control malaria.

## Background

Malaria remains a public health problem with the highest burden in the African Region of the World Health Organization (WHO). Globally, in 2022, there were 249 million malaria cases, and the WHO African Region accounted for 94% of the cases [1]. This same report also indicated that Nigeria (27%), the Democratic Republic of Congo (12%), Uganda (5%) and Mozambique (4%) accounted for almost half of the total global malaria cases [1].

In Uganda, malaria is the foremost cause of morbidity and mortality, and a recent publication acknowledged that it led to 29.1% outpatient visits, 39.5% inpatient admissions, and 10.9% hospital deaths [2]. Looking into the age group of children under 5 years of age, the national prevalence of malaria was reported at 9% between December 2018 and February 2019, with *Plasmodium falciparum* accounting for 97%, followed by *Plasmodium malariae* (10%), *Plasmodium ovale* (2%) and mixed infection (9%) [3]. Scarce data exist in Uganda about prevalence of malaria among children under 15 years old and yet according to a 2023 Malaria Consortium report, children aged 5–15 years are at risk of malaria and often without associated symptoms contributing to further parasite transmission and hindering elimination efforts [4].

Several studies have also been conducted by scholars within the country among children under 5 years in recent years; a 76% prevalence of malaria among children under five years of age was reported in two general hospitals in Karamoja [2], and another study reported a 23.3% prevalence of malaria among children under five years of age in the whole country on the basis of evidence from the previous 2018–2019 Uganda Malaria Indicator Survey [5]. Despite the malaria control measures put by the Ministry of Health, such as the distribution and use of insecticide-treated mosquito nets, intermittent preventive treatment of malaria during pregnancy and indoor residual spraying, malaria remains a public health challenge among children in Uganda [3]. The utilization rate of long lasting insecticide treated net (LLITN) in Africa remains considerably low, although a small segment of the population uses them regularly. The key reasons for not using nets include discomfort from heat and concerns about the chemicals they contain [6, 7].

Furthermore in Uganda, there is limited research on the impact of changing weather patterns, such as increased rainfall and temperature variations, on malaria vectors and transmission dynamics and Uganda shares porous borders with Kenya, Tanzania, South Sudan, and Rwanda, all of which experience significant malaria burdens and cross-border transmission of malaria is possible. The 2018/2019 Uganda Malaria Indicator Survey also indicated a decline in the markers of malaria infection and disease by 30%, but a gap was noted in the declines since it was not uniform all through the country.

Bugisu sub-region is a moderate to high malaria burden area with prevalence of malaria among children under 5 years of age reported at 5% [3]. Bududa General Hospital observed an increase in malaria infection among children under 15 years between April and June, 2023. However, data about prevalence of malaria and associated factors among children under 15 years are not well documented and yet children aged 5–15 years has been shown to be at risk of asymptomatic malaria infection and this affects elimination and control targets. This study set forth information to guide intervention among children at risk both in moderate and high transmission areas of Uganda. Therefore, this study aimed to determine the prevalence of malaria and associated factors among children under 15 years of age at Bududa General Hospital, Eastern Uganda, and address the above knowledge gaps.

## Methods

### Study site

This was a cross-sectional study carried out at Bududa General Hospital, a public healthcare institution in the district of Bududa in the Bugisu sub-region in Eastern Uganda. It is located approximately 38 km southeast of Mbale city. The coordinates of Bududa General Hospital are 01° 00′ 34.0″ N latitude and 34° 19′ 58.0″ E longitude. The hospital has 100 beds and offers a variety of medical services, including inpatient and outpatient treatment, maternity care, surgical services, laboratory services, and pharmacy services.

Bududa District lies on the slopes of Mount Elgon, and the climate is relatively humid montane. The district's altitude ranges from 1250 to 2850 m, with the highest points reaching 3000 to 4000 m at the peak of Mount Elgon. The district's rainfall is highly dependent on altitude, with higher altitudes receiving more rainfall than lower areas. Some of the harsh climatic conditions in Bududa District include: increased rainfall days, intense rainfall, rising temperatures, unreliable rainfall, and increase in windstorms. These conditions have led to an increase in landslides, rock falls, mudslides, and flooding.

The area experiences bimodal rainfall, with two rainy seasons (March to May and September to November) with annual rainfall ranges between 1200 and 1800 mm, average temperatures range from 16 °C to 25 °C, high humidity levels and floods. This climate has favoured the survival and reproduction of *Anopheles funestus* a major vector in rural areas of Uganda, which prefers permanent water bodies with vegetation, such as slow moving streams and ponds.

### Study design

This cross-sectional study was conducted between April 2023 and June 2023. The choice of this study period was primarily the increased number of malaria cases among children at Bududa General Hospital in the above mentioned months, which could be influenced by the climatic conditions of Bududa District and neighbouring areas since previous malaria surveys were conducted between December 2018 and February 2019.

### Study population

Febrile children below 15 years at Bududa General Hospital.

#### Inclusion criteria

- Age below 15 years
- Fever ≥ 37.5 °C in the past 48 h
- Parents/guardians provided written informed consent for participation.

#### Exclusion criteria

- Non-febrile children
- Children with chronic or congenital illnesses (e.g., sickle cell disease, HIV/AIDS)
- Children already receiving anti-malarial treatment
- Children with recent malaria diagnosis (e.g., within the last two weeks)
- Severe acute illness (e.g., bacterial infections, viral illnesses).
- Children on long-term prophylaxis for malaria.

### Sample size calculation

The required sample size for enrolment was estimated the Kish Leslie formula for a single population proportion on the following assumptions: a malaria prevalence of 19.7%, a 95% confidence level, and a 5% marginal error. This estimation was informed by a study conducted in Uganda [8]. The formula for calculating the sample size for proportion was as follows:

$${\text{n}} = \frac{{{\text{Z}}^{{2}} {\text{P }}\left( {{1} - {\text{ P}}} \right)}}{{{\text{d}}^{{2}} }}$$

where n = the desired sample size. Z = critical value of the normal distribution at 95%, which corresponds to 1.96. P = the proportion of the target population estimated to have malaria. d = estimated margin of error 5%. n = 243.

Hence, the calculated sample size was 243, but 250 study participants participated in this study.

### Sampling procedure

**Fig. 1:**
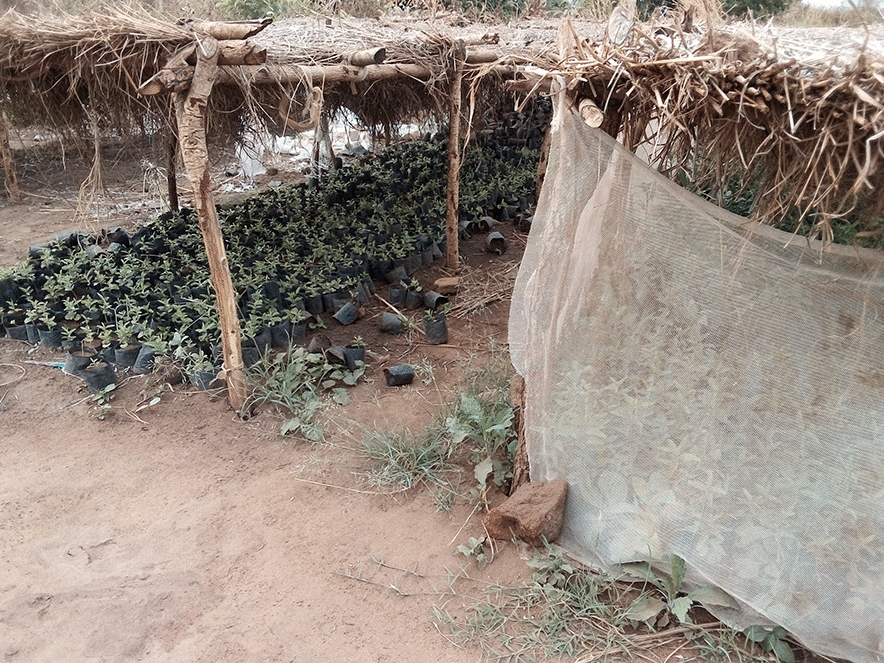
Insecticide treated mosquito nets being used to cover seedlings in a nursery bed

**Fig. 2:**
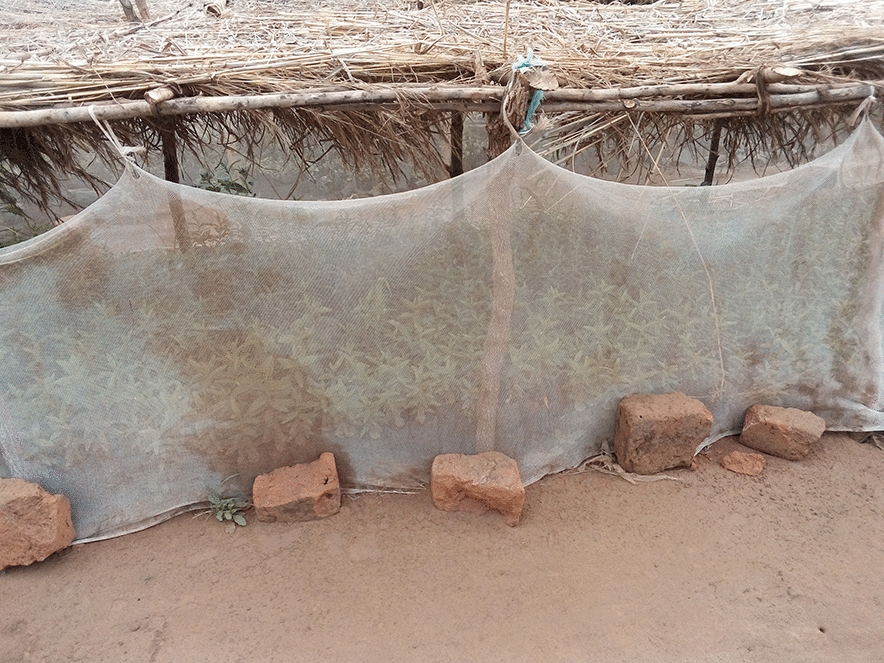
Insecticide treated mosquito nets being used to cover seedlings in a nursery bed

**Fig. 3:**
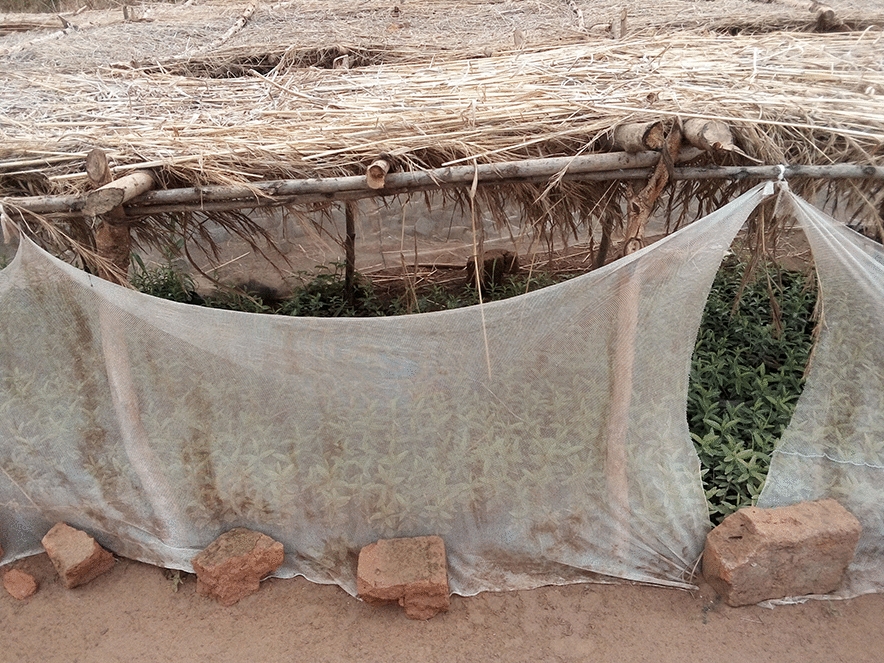
Insecticide treated mosquito nets being used to cover seedlings in a nursery bed

**Fig. 4:**
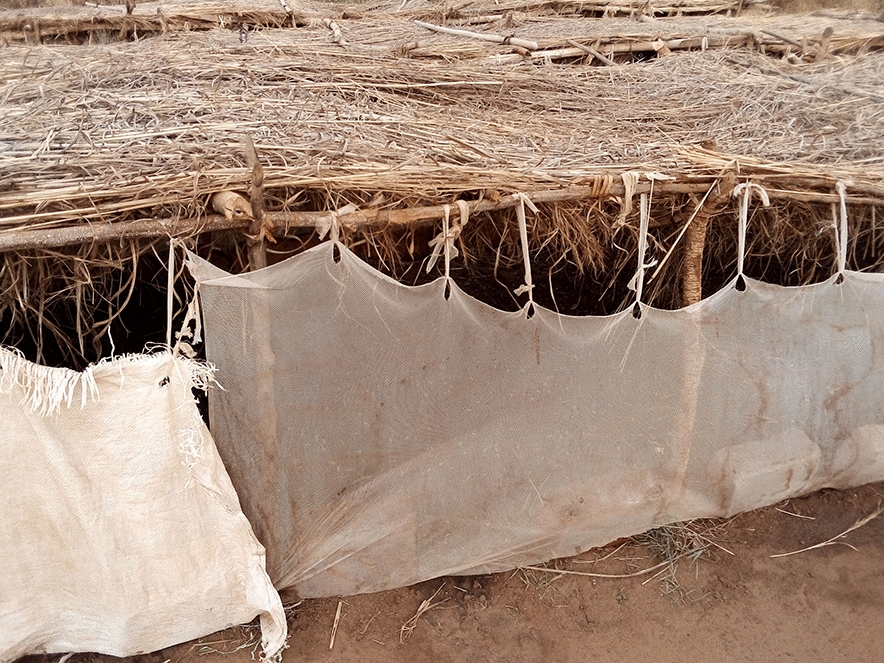
Insecticide treated mosquito nets being used to cover seedlings in a nursery bed

Bududa General Hospital was chosen because of its location in a malaria endemic area of Bugisu sub-region with its unique climate. Also the increase in the number of malaria cases between April to June, 2023 made it an ideal location to gather relevant data. The hospital could serve as a representative sampling point for understanding malaria trends in the broader community or district (Figs. 1, 2, 3, 4). In some communities, mosquitoe nets distributed  to prevent malaria are repurposed for other activities e.g fencing gradens, nursery beds, covering crops , all of which reduces their effectiveness for malaria prevention increasing their vulnerability to mosquitoe bites and malaria infections (Figs. 1, 2, 3, 4).

A simple random sampling method was used to recruit the participants, and those who declined were excluded. Participants were assigned folded papers with numbers written inside ranging from 1–40, and anyone who selected an even number was enrolled in the study until the calculated sample size was achieved.

### Data collection

Informed consent was obtained from the parents/guardians of the children who met the inclusion criteria, and a structured questionnaire was administered to the parents/guardians to collect their sociodemographic characteristics and identify factors associated with malaria. Approximately four millilitres (4 ml) of venous blood samples were collected from the children in an ethylene-diamine-tetraacetic acid (EDTA) vacutainer and screened for the presence of malaria parasitaemia using blood smear microscopy method. Thick and thin blood smears were prepared, stained with Giemsa and examined at × 100 magnification for malaria parasite detection, species identification and parasite counting. Parasite density was determined by counting the number of malaria parasites present and the number of white cells present simultaneously. Counting was stopped when ≥ 100 parasites in 200 white cells were counted or if ≤ 99 parasites in 500 white cells were counted. The parasite density was then calculated by dividing the number of parasites by the number of white cells counted and multiplying by 8000 and reported as parasite/µL of blood according to WHO guidelines [9].

The parasite density was classified as mild (parasite number = 5–10,000/µL of blood), moderate (parasite number = 10,000–100,000/µL of blood) or severe (parasite number > 100,000/µL of blood) [10]. Participants with positive results were referred to physicians for management.

### Quality control

A standard operating procedure (SOP) was employed and followed at each step of the testing process to ensure data quality. Structured questionnaires and a checklist were pretested for appropriateness. The Giemsa stain working solution was freshly prepared daily. Furthermore, the glass slides were labelled with the study participants’ unique code numbers. A sterile, single-use syringe was used for each child during blood sample collection, and the smears were reviewed by a WHO-certified malaria microscopist.

### Data analysis

The data collected were entered into an Excel spreadsheet and analysed using STATA software version 14.0. Categorical data and continuous variables are presented using descriptive statistics in the form of percentages, frequencies and pie-chart. Associations between malaria prevalence and factors were determined using bivariate logistic regression analysis. Factors with p values less than or equal to 0.1 in the bivariate analysis were subjected to multivariate logistic regression analysis. Factors with p values ≤ 0.05 were considered statistically significant.

### Ethical consideration

This study was approved by the Faculty Research Committee (FRC), Faculty of Medicine, Mbarara University of Science and Technology, and the office of the hospital director, Bududa General Hospital (approval number MUST/MLS/030). Informed consent was obtained from the parents/guardians of the children, and they were assured of confidentiality. The purpose of the study was explained to them that their participation was free and voluntary and that they were free to withdraw from the study at any time and would not affect their access to medical attention.

## Results

### Demographic characteristics of the study participants

This study enrolled 250 study participants. The mean age was 6.2 years (SD = 4.95 years). The majority of the study participants were aged between 1 and 5 years (37.6%), were females (54%), were living in rural areas (89.6%), had parents/guardians who had attained secondary education (57.2%), and peasant farmers (72%), as shown in Table 1.

**Table 1:** Socio-Demographic Characteristics of study Participants

Variables		Frequency (n)	Percentage (%)
Age (years)	< 1	36	14.4
	1–5	94	37.6
	6–10	53	21.2
	11–15	67	26.8
Sex	Male	115	46.0
	Female	135	54.0
Residence	Rural	224	89.6
	Urban	26	10.4
Education level of parent/guardian	Primary	100	40
	Secondary	143	57.2
	Tertiary	7	2.8
Occupation of parent/guardian	Business	21	8.4
	Peasant	180	72
	Others	49	19.6
Household income	< Ugx. 100,000/ =	107	42.8
	≥ Ugx. 100,000/ =	143	57.2
Number of children in household	≤ 4	227	90.8
	> 4	23	9.20
Distance to hospital	≤ 2 km	99	39.6
	> 2 km	151	60.4

Ugandan Shillings (Ugx)

### Prevalence of malaria

The overall prevalence of malaria was 111 (44.4%) among children aged less than 15 years at Bududa General Hospital. Among the children who tested positive for malaria, 98 (88.3%) had *P. falciparum*, 11 (9.9%) had *P. malariae* and 2 (1.8%) had *P. ovale* infection. The mean parasite count was 21,951 parasites/µL of blood, the highest parasite count was 154,387 parasites/µL of blood, and the lowest count was 146 parasites/µL of blood. The prevalence rates of mild, moderate, and severe malaria parasitaemia among children with malaria were 52 (46.8%), 31 (28.0%), and 28 (25.2%), respectively.

### Factors associated with malaria infection

The associations between malaria prevalence and various factors (age, sex, education level of parents/guardians, use of insecticide-treated mosquito nets, stagnant water around the home, household income, number of children in the household, type of housing, residence, occupation of parents/guardians, history of malaria infection, and staying outside at night) were assessed using logistic regression analysis. In the bivariate analysis, older age (1–5 years (p = 0.008), 6–10 years (p = 0.000), and 11–15 years (p = 0.000)) and not using insecticide-treated mosquito nets (p = 0.001) were significantly associated with malaria (Table 2).

**Table 2:** Bivariate analysis of factors associated with malaria

Variables		Odds ratio	95% Confidence interval	P value
Age (years)	< 1	Ref		
	1–5	4.01	1.43–11.27	0.008*
	6–10	10.79	3.63–32.06	0.000*
	11–15	6.93	2.38–20.23	0.000*
Sex	Male	Ref		
	Female	1.15	0.64–2.06	0.640
Occupation	Business	Ref		
	Peasant	0.88	0.28–2.76	0.830
	Others	0.94	0.28–3.15	0.920
Traveling history	No	Ref		
	Yes	0.69	0.27–1.74	0.427
Residence	Rural	Ref		
	Urban	1.66	0.57–4.84	0.351
Number of household children	< 4	Ref		
	≥ 4	0.79	0.29–2.16	0.642
Distance to hospital	≤ 2 km	Ref		
	> 2 km	0.79	0.42–1.48	0.462
Housing	Permanent with screen	Ref		
	Permanent without screen	0.30	0.06–1.49	0.140
	Semi-permanent with screen	0.84	0.31–2.28	0.736
	Semi-permanent without screen	0.33	0.11–1.04	0.056
Education level	Primary	Ref		
	Secondary	1.73	0.89–3.37	0.107
	Tertiary	0.71	0.09–5.70	0.747
Household income (Ugandan shillings)	< 100,000/ =	Ref		
	≥ 100,000/ =	0.63	0.33–1.20	0.161
Stay outside at night	No	Ref		
	Yes	1.36	0.64–2.86	0.424
Stagnant water	No	Ref		
	Yes	1.48	0.41–5.33	0.549
Use of Insecticide treated mosquito net	Yes	Ref		
	No	3.78	1.75–8.12	0.001*

*Significant

According to multivariate analysis, individuals aged 1–5 years (p = 0.013), 6–10 years (p = 0.000), and 11–15 years (p = 0.000) had a greater risk of malaria infection than younger individuals did (< 1 year). Additionally, children of parents/guardians who had attained secondary education (p = 0.050) were at greater risk of malaria infection than were children of parents who had attained primary education. Finally, children who were not using insecticide-treated mosquito nets (p = 0.002) were at greater risk of suffering from malaria than were children who were sleeping under insecticide-treated mosquito nets (Table 3).

**Table 3:** Multivariate analysis of factors associated with malaria

Variables		Adjusted odds ratio	95% Confidence interval	P value
Age (years)	< 1	Ref		
	1–5	3.47	1.30–9.27	0.013*
	6–10	9.60	3.35–27.60	0.000*
	11–15	6.34	2.30–17.49	0.000*
Education level	Primary	Ref		
	Secondary	1.84	1.00–3.37	0.050*
	Tertiary	1.10	1.18–6.63	0.919
Insecticide treated mosquito net usage	Yes	Ref		
	No	2.93	1.48–5.84	0.002*
Household income (Ugandan shillings)	< 100,000/=	Ref		
	≥ 100,000/=	0.62	0.34–1.12	0.110

*Significant factor

## Discussion

The prevalence of malaria among febrile children under 15 years of age in the present study was 44.4%, with prevalences of mild, moderate, and severe malaria of 46.8%, 28.0%, and 25.2%, respectively. The overall prevalence of malaria revealed in this study differs from the findings of the Uganda Malaria indicator health survey, which reported that the prevalence of malaria among children under 5 years of age was 9% and 5% in the Bugisu region [11], and other Ugandan researchers reported 19.04% [12], 19.7% [8], 23.3% [5] and 76% [2] prevalence rates of malaria among children under 5 years of age. The possible explanations for this observation could be differences in age specifics. The Uganda Malaria Indicator Health Survey focused on children under 5 years, while our study examined children under 15 years. Other reasons of the differences in findings could be the access to and use of insecticide-treated mosquito nets, differences in the malaria peak for different regions in Uganda, differences in vector density due to rainfall patterns and temperatures, changes in biting patterns, which are now both indoors and outdoors, parasite variations (*P. falciparum, P. malariae* and *P. ovale*), drug resistance, the type of housing and vegetation density and all these factors influence mosquito breeding, infective mosquito bites, and malaria infection recurrence or recrudescence.

This study's findings were also incompatible with previous findings, which reported prevalences of 12.2% [13] and 14% [14], respectively, in Rwanda. Several studies reported rates of 24.6% [15], 22.1% [16], 14.7% in Ethiopia [17], and 33% in Malawi [18]. The variation in prevalence could be due to diagnostic criteria, sample size differences, geographical disease distribution, study design, health-seeking habits, and the socioeconomic status of these children's parents. This current findings, however, were closely comparable to the reported prevalence rates of malaria of 36.6% in Uganda [19], 44% in Guinea [20], and 37% in Malawi [21].

*Plasmodium falciparum* infection was the most common malaria infection among the children in the current study. Other species reported in the present study also include *P. malariae* and *P. ovale* infections. This finding is in line with reports from the Uganda Malaria Indicator Health survey, which also reported *P. falciparum* infection to be the most common malaria infection, followed by *P. malariae* and *P. ovale* infections, among children [3].

This study also revealed that older age was associated with malaria infection and that children who were older were more likely to suffer from malaria. Although participants with fever were recruited, the above finding is comparable with two studies conducted in Uganda, which reported a similar finding that older age is a predictor of malaria among children under 15 years of age [5, 12], and in Kenya [22]. This observation has been attributed to the age related immunity that develops in these children because of continuous exposure to infective mosquito bites, and this immunity develops first against complicated malaria, then to uncomplicated malaria and to malaria parasitaemia, explaining the high prevalence of malaria parasitaemia among older children but without them developing clinical disease [23]. The Malaria Consortium report highlighted the emerging challenges of global malaria trends as being changing epidemiological patterns with shifts in at risk populations being children aged 5–15 years who are likely to have the malaria infection without associated symptoms and also less likely to use mosquito nets and receive treatment [4].

This study also reported that parents’/guardians’ secondary level of education was associated with high malaria parasitaemia. This finding is not in agreement with a finding of a study in Uganda that revealed an association between the primary and tertiary education levels of the child’s parents or guardians and a lower risk of malaria infection [12]. Other studies were also not in agreement with this finding about parents’/guardians’ education [21, 24]. However, parent/guardian education attainment is associated with higher socioeconomic status, a wealthier household, and a better life, which leads to a reduced risk of developing malaria infection [12]. Parents and guardians who had not received health education in the past 6 months were more likely to have children suffering from malaria due to the knowledge gap in disease control and prevention [15], which still elaborates on the significance of parent/guardian education in influencing their children’s health.

Children who were not sleeping under insecticide-treated mosquito nets were 2.93-fold more likely to develop malaria parasitaemia than were those who were sleeping under insecticide-treated mosquito nets. This finding was in line with a study that reported a 15-fold increase in the risk of developing malaria among children who were not sleeping under insecticide-treated mosquito nets [13]. This study was also in agreement with studies that linked malaria infection with irregular utilization of insecticide-treated bed nets, and the probable explanation was continuous exposure to infective mosquito bites that can lead to malaria infection [15, 24]. 

## Limitation of the study

This study employed a cross-sectional design and was conducted in a hospital setting, which limits its generalizability to other regions. Additionally, the research was carried out between April and June 2023, whereas the peak malaria transmission period in Uganda typically occurs from November to January.

## Conclusion

On the basis of the study results, malaria remains a significant public health issue, with an overall prevalence of 44.4%, with almost half of the study participants having a severe form of malaria. Age, the mother's education level, and failure to use insecticide-treated bed nets were identified as significant factors associated with malaria infection. These findings underscore the need for targeted interventions, such as promoting the use of bed nets and increasing awareness of malaria prevention, particularly in vulnerable age groups and less educated populations.

## Data Availability

No datasets were generated or analysed during the current study.

## Abbreviations

WHO: World Health Organization
LLITN: Long lasting insecticide treated net
EDTA: Ethylene diamine tetra acetic acid
µL: Microlitre
FRC: Faculty research committee
SOP: Standard operating procedure
MUST: Mbarara University of Science and Technology
MLS: Medical laboratory science
HIV: Human immunodeficiency virus
AIDS: Acquired immunodeficiency syndrome
SD: Standard deviation
*P. falciparum*: *Plasmodium falciparum*
*P. malariae*: *Plasmodium malariae*
*P. ovale*: *Plasmodium ovale*
